# Unintentional injury mortality among children under age five in urban and rural areas in the Sichuan province of west China, 2009–2017

**DOI:** 10.1038/s41598-019-38936-6

**Published:** 2019-02-27

**Authors:** Minghong Yao, Gonghua Wu, Ziling Zhao, Min Luo, Juying Zhang

**Affiliations:** 1 Department of Epidemiology and Health Statistics, West China School of Public Health, Sichuan University, Chengdu, Sichuan People’s Republic of China; 2 Sichuan Provincial Maternal and Child Health Hospital, Chengdu, Sichuan People’s Republic of China

## Abstract

This study analysed trends in the unintentional injuries specific mortality rates among children under age five (UI-specific U5MRs) in urban and rural areas in the Sichuan province of western China. Data were obtained from the National Health Statistics Survey System. The Cochran-Armitage trend test was used to analyse the trends in UI-specific U5MRs and the proportion of unintentional injury deaths to total deaths. The Poisson regression model was used to compare the UI-specific U5MRs between rural and urban areas. The overall UI-specific U5MRs decreased from 3.8 to 1.7 per 1,000 live births from 2009 to 2017, with an average annual decline in the rates of 8.78% and 10.05% in urban and rural areas, respectively. The UI risk of death in rural areas was approximately 1.95 times that in urban areas (95% CI: 1.73–2.18; *p* < 0.01). A total of 49.9% of all the children in the study did not receive any treatment before death caused by UI. The UI-specific U5MRs significantly declined in Sichuan province from 2009 to 2017, but large disparities in UI-specific U5MRs in urban and rural areas still exist. Reducing the U5MRs due to UI should be a major public health concern in western China.

## Introduction

The mortality rates among children under age five (U5MRs) is one of the most important indicators reflecting the development of a country’s healthcare system; therefore, reducing the U5MRs is a priority for all countries in the world. The United Nations (UN) set Millennium Development Goal (MDG) 4 to reduce the U5MRs by two-thirds between 1990 and 2015; however, only 62 countries have achieved that goal^1^. To further improve the health of children, the UN proposed the Sustainable Development Goals (SDG) in 2015, and the third goal is to eliminate preventable deaths among new-borns and children under age five by 2030^2^. However, even if all countries meet the SDG 3, 56 million children are still projected to die by 2030^1^. Therefore, improving the health of children and reducing children’s mortality still require more sustained efforts by the government. To effectively reduce the U5MRs, it is necessary to analyse the causes of death of children.

Unintentional injuries (UI) have become a hot topic in the field of public health because of their high disability and mortality^3^. Compared with adults, children are a high-risk group for UI^4^. Liu *et al*. found that child deaths caused by UI is one of the slowest declines from 2000 to 2013, and approximately 0.324 million children died caused by UI in 2013^5^. UI have become the leading cause of death for children aged 0 to 14 in the world^6^. China is one of the few countries that has achieved MDG 4; however, the absolute number of deaths among children under age five each year is substantial due to the large population base^7^. UI are the third leading cause of death for children under age five and the first leading cause of death for children aged 1–5 years in China^7^. These figures warn us that prevention and control of children’s deaths caused by UI is imperative, and they are key measures for further reducing the overall U5MRs in China.

The U5MRs in the western regions of China is still higher than in the eastern and central regions due to differences in economic development and natural conditions^7^. The effective reduction of U5MRs in western regions will play an important role in reducing the overall U5MRs in China. As the most populous province in the western region, Sichuan plays a key role in reducing the U5MRs in western regions. In this article, we describe the profile of UI-specific U5MRs in Sichuan province from 2009 to 2017, analysing the main causes of UI among children under age five. These findings may help policy-makers make well-informed decisions to formulate reasonable measures to prevent UI, which could eventually effectively reduce U5MRs.

## Method

### Variables and data source

The classification of UI death (The International Classification of Diseases, Revision 10, ICD-10: V01-X59) includes drowning (ICD10: W65-W74), traffic accidents (ICD10: V01-V98), accidental asphyxia (ICD10: W75-W84), accidental poisoning (ICD10: X44-X49), accidental falls (ICD10: W00-W19) and other accidental deaths (ICD10: W20-W64, W85-W94, X00-X43, X50-X59). The UI-specific U5MRs (per 1,000 live births) was calculated as the number of deaths from UI divided by the number of live births within the same period. The proportion of unintentional injury deaths refers to the proportion of deaths directly caused by unintentional injuries among the total number of deaths among children under age five. Infants were defined as children who were younger than 1 year old; neonates were infants within the first 28 days after birth; post-neonates were infants who were older than 28 days and had not celebrated their first birthday; and children were defined as those who were aged 12 months to 59 months. Data for this study were obtained from the National Health Statistics Survey System. Determination of causes of child mortality and quality control of the data were the same as in the He *et al*. study^8^.

### Ethics approval and consent to participate

The ethics approval and consent to participate of this study is dispensable for the following reasons: first, the data used in this study were derived from the National Health Statistics Survey System, which is derived from the official report cards approved by the government in China and no longer need separate ethical approval and informed consent. Second, the information was encrypted according to the national requirements and was directly submitted to the National Health Statistics Survey System through the network. Therefore, no one could obtain the children’s personal information such as name, identity card number, address, telephone numbers and so forth. Third, the Sichuan government publishes child death data on the official websites every year (http://www.scwst.gov.cn/xx/tjxx/tjnj/). Finally, our research focuses on the overall levels in urban and rural areas rather than on individual subpopulations.

### Statistical analysis

The Cochran-Armitage trend test was used to analyse the trends in UI-specific U5MRs and the proportion of unintentional injury deaths to total deaths. The Poisson regression model was used to compare the UI-specific U5MRs between rural and urban areas. SAS 9.3 software was used for all analyses. A *p* value < 0.05 was used to define the level of significance.

## Result

### Trends and composition of the UI-specific U5MRs

**Figure 1:**
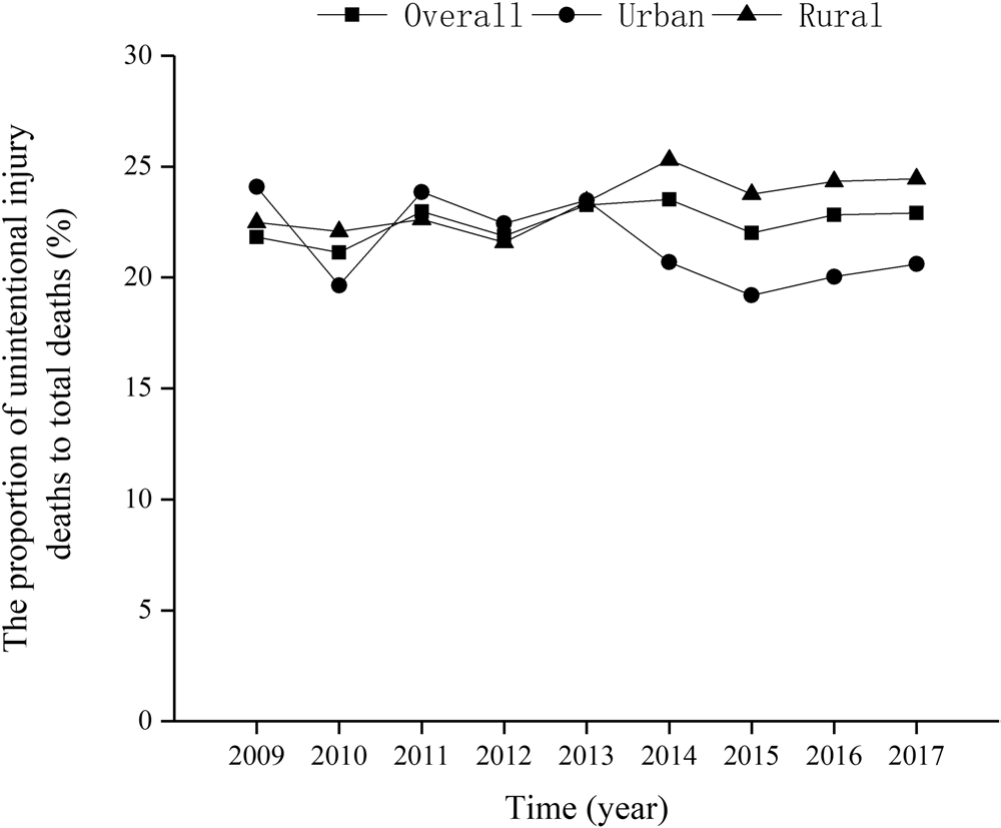
The proportion of UI deaths to total deaths among children under age five in Sichuan province of Western China.

**Figure 2:**
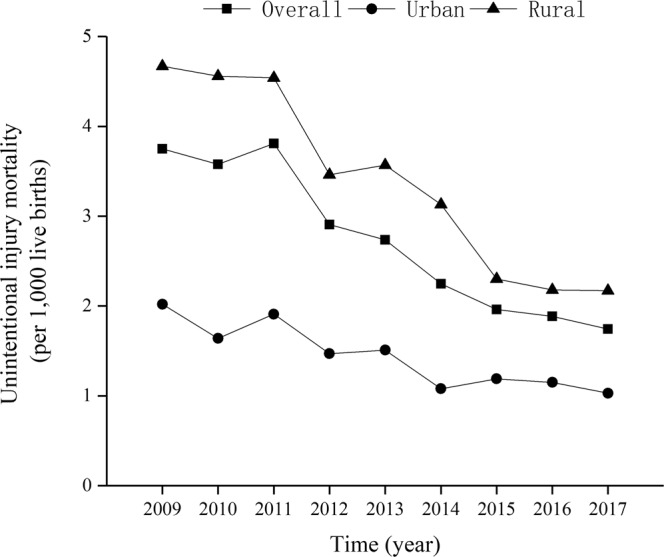
The trend of UI-specific U5MRs in urban and rural areas in Sichuan province of Western China.

The overall proportion of UI deaths to total deaths increased from 21.8% in 2009 to 22.9% in 2017 (${\chi }_{trend}^{2}$ = 15.37, *p* < 0.01) (Fig. 1). The overall UI-specific U5MRs decreased from 3.75 per 1,000 live births in 2009 to 1.74 in 2017 (${\chi }_{trend}^{2}$ = 2679.54, *p* < 0.01), and the annual rate of decline was 10.07% (Fig. 2). The UI-specific U5MRs also significantly decreased from 2009 to 2017 in both urban (decreased by 49.00%; ${\chi }_{trend}^{2}$ = 431.23, *p* < 0.01) and rural (decreased by 53.53%, ${\chi }_{trend}^{2}$ = 1718.62, *p* < 0.01) areas; the annual rates of decline were 8.78% and 10.05%, respectively (Fig. 2).

From 2009 to 2017, the proportions of UI deaths to total deaths in neonates were significantly decreased (${\chi }_{trend}^{2}$ = 21.57, *p* < 0.01), but the proportions of UIs deaths to total deaths both in post-neonates (${\chi }_{trend}^{2}$ = 4.45, *p* = 0.04) and children (${\chi }_{trend}^{2}$ = 28.68, *p* < 0.01) were significantly increased. The UI-specific rates decreased by 69.39% (${\chi }_{trend}^{2}$ = 579.04, *p* < 0.01), 21.69% (${\chi }_{trend}^{2}$ = 199.99, *p* < 0.01), and 57.83% (${\chi }_{trend}^{2}$ = 2878.96, *p* < 0.01), respectively, in neonates, post-neonates and children (Table 1).

**Table 1:** The difference of unintentional injury mortality (per 1000 live births) and proportion (%) of UI deaths to total deaths of neonates, post-neonates and children, 2009–2017.

Year	Neonates		Post-neonates		Children	
	UIM	Proportion	UIM	Proportion	UIM	Proportion
2009	0.49	6.45	0.83	18.61	2.49	48.76
2010	0.47	6.28	0.81	17.95	2.33	47.41
2011	0.47	6.37	0.91	20.70	2.31	47.96
2012	0.40	5.60	0.63	21.18	1.49	48.23
2013	0.36	5.53	0.50	18.93	1.37	50.88
2014	0.29	5.19	0.50	21.39	0.77	49.13
2015	0.22	4.98	0.60	17.80	0.55	49.43
2016	0.18	5.36	0.50	19.30	1.13	50.06
2017	0.15	4.72	0.65	20.63	1.05	51.46
*χ* ^*2*^	579.04	21.57	199.99	4.45	2878.96	28.68
*P*_*trend*_	*P* < *0.01*	*P* < *0.01*	*P* < *0.01*	*0.04*	*P* < *0.01*	*P* < *0.01*

Note: UIM, unintentional injuries mortality rate.

### Comparison of the UI-specific U5MRs between rural and urban areas and other factors

Table 2 shows the results of Comparison of the UI-specific U5MRs between rural and urban areas and other factors. The risk of unintentional injury death among male children was 1.15 times that of female children (95% CI: 1.03–1.29; *p* = 0.02). As the children’s age increased, the risk of unintentional injury death among children under age five was significantly increased (RR: 2.13, 95% CI: 2.00–2.28; *p* < 0.01). The unintentional injury death risk in rural areas was approximately 1.95 times that in urban areas (95% CI: 1.73–2.18; *p* < 0.01).

**Table 2:** The result of Comparison of the UI-specific U5MRs between rural and urban areas and other factors.

Variable	Β (95%CI)	SE	RR (95%CI)	Wald χ^2^	P
Sex	0.14 (0.03, 0.26)	0.06	1.15 (1.03, 1.29)	5.88	0.02
Age	0.76 (0.69, 0.83)	0.03	2.13 (2.00, 2.28)	494.58	<0.01
Year	−0.03 (−0.05, −0.01)	0.01	0.97 (0.95, 0.99)	9.92	<0.01
UR	0.67 (0.55, 0.78)	0.06	1.95 (1.73, 2.18)	129.45	<0.01
Sex*Age	0.14 (0.09, 0.19)	0.03	1.15 (1.09, 1.21)	26.74	<0.01
Sex*Year	−0.01 (−0.02, 0.01)	0.01	1.00 (0.98, 1.01)	0.13	0.72
Sex*UR	−0.02 (−0.09, 0.06)	0.04	0.98 (0.91, 1.06)	0.23	0.63
Age*Year	0.02 (0.01, 0.03)	0.01	1.02 (1.01, 1.03)	12.99	<0.01
Age*UR	0.04 (−0.01, 0.10)	0.03	1.04 (0.99, 1.10)	2.31	0.13
Year*UR	−0.02 (−0.03, −0.01)	0.01	0.98 (0.97, 1.00)	6.13	0.01

Note: *β*, regression coefficient; CI: confidence interval; UR, urban-rural; SE, standard error; RR: relative risk.

### Causes of UI

Table 3 shows that drowning and accidental asphyxia were the main causes of UI in Sichuan, with overall proportions of 36.4% and 31.7%, respectively. Table 4 shows the causes of UI death among children of different areas, sex, and ages. Although the proportions of causes of death in urban and rural areas are different (*χ*^2^ = 46.72, *P* < 0.01), drowning and accidental asphyxia are still the main causes of UI deaths, with 37.75% and 30.58% in urban areas and with 33.70% and 33.84% in rural areas, respectively. When divided into three periods (neonates, post-neonates and children), the proportions of causes of UI death are different (*χ*^2^ = 6678.53, *P* < 0.01), with accidental asphyxia being the leading cause among neonates and post-neonates (85.8% and 71.6%, respectively) and drowning being the leading cause for children (53.8%).

**Table 3:** The proportion of UI death causes among total unintentional injuries deaths (%), 2009–2017.

Year	Drowning	Accidental asphyxia	Traffic accidents	Accidental falls	Accidental poisoning	Others	Total
2009	41.7	29.0	10.2	5.8	3.2	10.1	100.0
2010	37.0	31.0	13.0	6.0	4.1	8.9	100.0
2011	37.8	31.8	12.1	5.6	3.5	9.2	100.0
2012	36.7	33.1	13.4	6.1	2.7	8.0	100.0
2013	35.8	29.9	16.1	6.1	3.0	9.1	100.0
2014	36.4	34.0	13.7	5.5	2.5	7.9	100.0
2015	36.3	31.0	14.4	7.7	2.7	7.9	100.0
2016	33.1	33.2	17.1	7.5	2.2	6.9	100.0
2017	33.1	31.9	16.7	9.2	2.2	6.9	100.0
Overall	36.4	31.7	14.1	6.5	2.9	8.4	100.0
*χ* ^*2*^	684.32	111.60	1045.61	387.11	273.37	361.47	—
*P*_*trend*_	*P* < *0.01*	*P* < *0.01*	*P* < *0.01*	*P* < *0.01*	*P* < *0.01*	*P* < *0.01*	—

**Table 4:** The difference of proportions of UI death causes for children under age five (*n*, %).

Factors	Causes of UIs deaths						*χ* ^2^	*P*
	Drowning	Accidental asphyxia	Traffic accidents	Accidental falls	Accidental poisoning	Others		
**Areas**								
Urban	3154 (37.8)	2555 (30.6)	1122 (13.4)	518 (6.2)	258 (3.1)	749 (8.9)	46.72	<0.01
Rural	1440 (33.7)	1446 (33.8)	658 (15.4)	308 (7.2)	113 (2.6)	308 (7.2)		
Gender*								
Male	2888 (55.9)	445 (8.6)	878 (17.0)	421 (8.2)	160 (3.1)	375 (7.3)	38.17	<0.01
Female	1574 (50.4)	259 (8.3)	655 (21.0)	242 (7.7)	129 (4.1)	266 (8.5)		
**Age**								
Neonates	16 (1.2)	1170 (85.8)	14 (1.0)	20 (1.5)	18 (1.3)	125 (9.2)	6678.53	<0.01
Post-neonates	115 (3.9)	2126 (71.6)	232 (7.8)	141 (4.8)	64 (2.2)	291 (9.8)		
Children	4463 (53.8)	705 (8.5)	1534 (18.5)	665 (8.0)	289 (3.5)	641 (7.7)		

Note: *, only 12-to-59 month-old children are included.

### The seasonal trend for the main causes of death in children and infants

**Figure 3:**
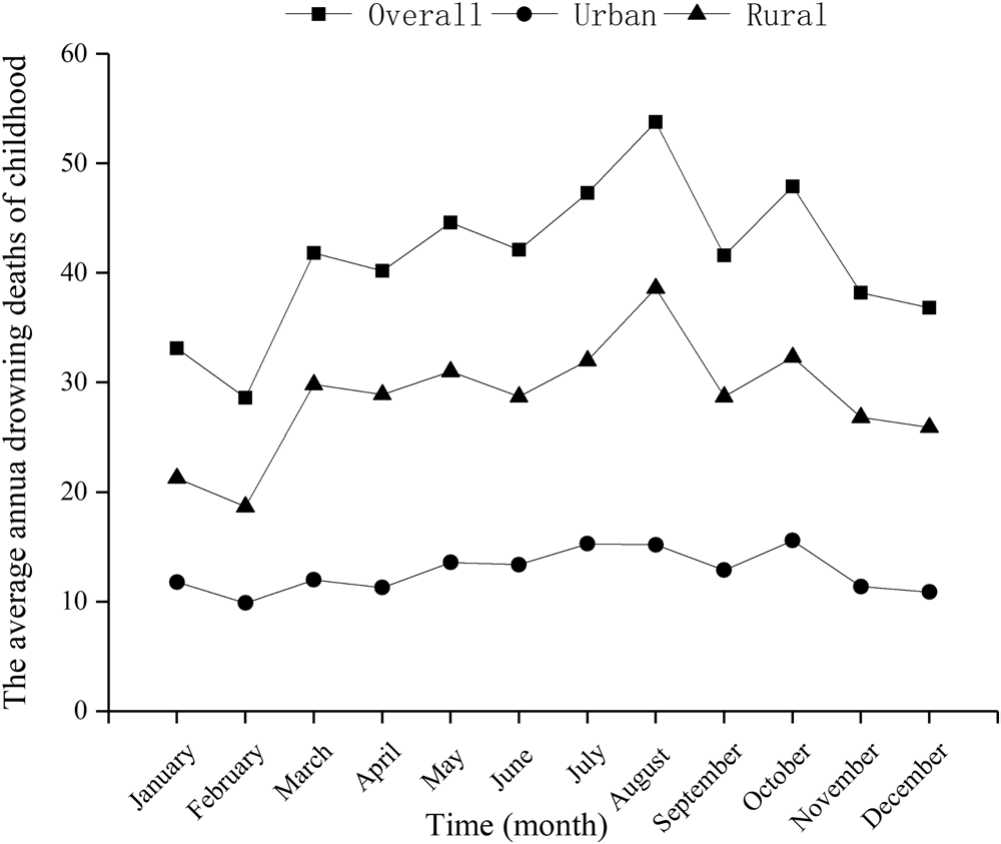
The average number of drowning deaths per month for children in urban and rural areas in Sichuan province of Western China.

**Figure 4:**
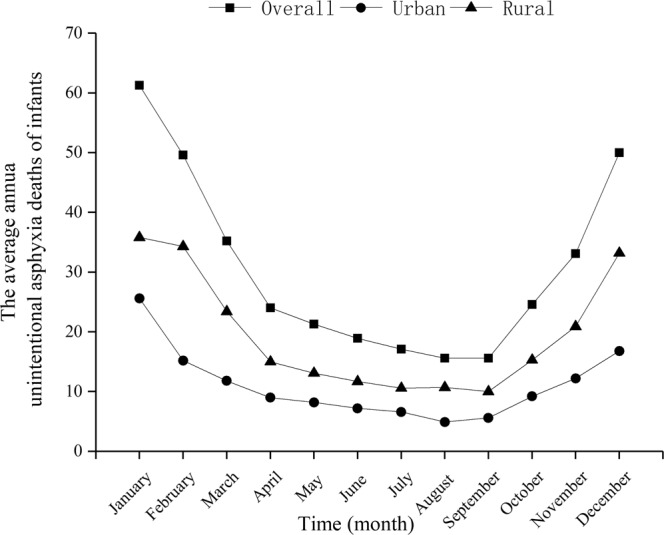
The average number of accidental asphyxia deaths per month for infants in urban and rural areas in Sichuan province of Western China.

Among children, the number of deaths in rural areas is highest in summer (August); however, the average number of children dying from drowning every month has changed little in urban areas (Fig. 3). Among infants, the average numbers dying from accidental asphyxia every month are highest in autumn (October and November) and winter (December, January and February) in both urban and rural areas (Fig. 4).

### Received Medical services before death for children who died from UI

**Figure 5:**
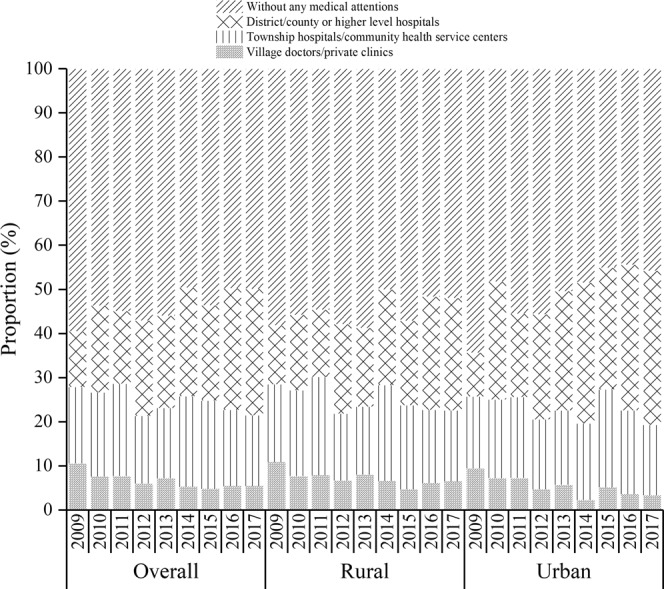
Proportions of children who sought medical services before death in urban and rural areas in Sichuan province of Western China.

Figure 5 shows the proportions of children who sought medical services before death from 2009 to 2017. The overall proportion of children who sought medical services at the district/county level or higher hospitals increased from 12.6% in 2009 to 28.6% in 2017, and the rate of increase in urban areas (from 9.9% in 2009 to 34.9% in 2017) was significantly higher than in rural areas (from 13.4% in 2009 to 25.5% in 2017). Although the proportion of children who did not receive any medical attention showed a downward trend, the proportion was still large in 2017 (overall: 49.9%, urban: 45.8%, rural: 51.9%).

## Discussion

In China, with the improvement of people’s living conditions and the development of medical and health care, children’s deaths caused by infectious diseases have been effectively controlled, and UI are gradually becoming the leading cause of death in children under age five^7,8^. Therefore, reducing UI-specific U5MRs is one of the keys to achieving SDG3 and effectively reducing U5MRs. During 2009–2017, the UI-specific U5MRs in Sichuan significantly decreased. Two reasons would stand for this improvement. On the one hand, with more attention paid to life safety after the big earthquake in 2008, security education concerning injury prevention and first aid greatly increased and has been carried out for a wide range of people with various ways^9^. Injury prevention education increases children’s recognition of safety, and there is less risk that children will sustain UI. On the other hand, many strategies have been implemented to improve children’s health conditions, and the declining mortality of neonates, post-neonates and children from 2009 to 2017 also tells the story. To effectively improve people’s health, China has implemented “The Basic Public Health Service Project”, and its per capita expenditure has been raised from 15 yuan in 2009 to 50 yuan in 2017. Regulations for child health management in “The Basic Public Health Services” stipulate that infants and young children must go to township hospitals or community health services at 3, 6, 8, 12, 18, 24, 30, and 36 months to receive health management services. The proportion of the health system involved in the management of children under 3 years, an indicator of health care accessibility, increased from 77.02% in 2009 to 94.34% in 2017^10,11^. At the same time, the Sichuan provincial government has also drawn up a document on improving children’s health, titled “The Outline for the Development of Children in Sichuan (2011–2020)”^12^. These measures directly or indirectly improve children’s health and reduce UI-specific U5MRs.

During 2009–2017, although UI-specific U5MRs declined both in urban and rural areas in Sichuan, the risk of UI deaths among children under age five in rural areas is still higher than in urban areas. In addition, the proportion of UI deaths to total deaths in rural areas has increased, but that proportion in urban areas has decreased from 2009 to 2017. This indicates that the overall situation regarding UI deaths among children is getting better, but the urban-rural disparity is still a challenge. These results were consistent with those that Jiang *et al*. and Wang *et al*. observed in other provinces in China^13,14^. Other countries in the world, such as India^15^, Ireland^16^, and South Africa^17^, have also found that the UI-specific U5MRs for children in rural areas is higher than in urban areas. As we know, the higher UI-specific U5MRs and proportion in rural areas was linked to the relatively poorer economy^18^, to lower levels of knowledge about prevention of UI^19^, and to allocation of medical resources^20^. In 2017, the per capita disposable income of urban residents in Sichuan was 2.51 times that of rural residents^21^. The education level of rural residents is generally lower than that of urban residents, and they have less access to safety education; this makes them less aware of UI prevention, and they cannot take effective measures to address UIs when a child sustains a UI. The uneven distribution of medical and health care resources is one of the reasons why the mortality of children in rural areas is higher than that in urban areas^22^; in many rural areas, especially in remote minority areas, the level of basic health facilities and medical services is relatively low and can only provide basic medical services; the average number of health care personnel was 4.21 per 1000 people in rural areas and 8.07 per 1000 people in urban areas in 2017.

The Poisson regression shows that boys are more likely to die from unintentional injuries than girls. In addition to the results of Yin *et al*. reported in 2015, the same relationship was found in Canada and India^23–25^. This may be because males are more physical actively and more likely to be exposed to UI risk factors. In addition, the risk of UI deaths in children increased with age. This was similar to the findings of He *et al*.^7^. One possible reason is that children’s ability to engage in activities and the scope of the activities increases gradually with increasing age, which increases the likelihood of exposure to UI risk factors. However, children’s ability to identify and avoid dangerous situations is limited, and thus it is easy for them to sustain a variety of UI events. The model also showed an interaction between sex and age in which older male children had a higher risk of unintentional injury death, suggesting that more attention should be paid to the prevention of unintentional injury death in older male children.

Only by analysing the cause of UI deaths can we provide a basis for formulating measures to prevent unintentional injury. During 2009–2017, drowning and accidental asphyxia were the main causes of UI-specific U5MRs both in urban and rural areas. The leading cause of unintentional deaths among the three age groups are different, with accidental asphyxia being the leading cause among neonates and post-neonates and drowning being the main cause in children, which is consistent with what Xu observed^26^. A possible reason is that most infants sleep in soft beds or sofas together with their parents, while some studies have shown that it could increase the risk of infants accidental asphyxia^27,28^. As age increases, children engage in increased physical activity and are curious about the outside world, increasing exposure to other risk factors such as for drowning and traffic accidents. In summary, to effectively reduce the number of deaths caused by UI in children, the government should strengthen the management of children at high risk of UI and pay more attention to accidental asphyxia in infancy and to drowning in children.

When children suffer from UI, if they can be treated in time, the survival rate will significantly improve. The results of this study show that 49.9% of children did not receive any treatment before death caused by UI. Part of the reason may be that the children suffered serious UI and died before they were found. The study also found that the proportion of children who did not receive any treatment in rural areas was higher than that in urban areas. The possible reason is that the travel is inconvenient in rural areas, and most of the residents are far from the nearest medical institutions; thus, the likelihood of receiving timely treatment in rural areas is lower than in urban areas^29^.

### Limitations

This study has some limitations. First, the UI death data were collected from the National Health Statistics Survey System. While the overall mortality rate is accurate, the proportions of different factors may differ from those of the population and may therefore affect the results. Second, we do not collect data on factors related to unintentional injury deaths in children, such as family economic level and parental education level; as a result, we cannot quantify these other risk factors for UI death in children. Because the denominator for UI-specific U5MRs per month is uncertain, we cannot explore the seasonality of UI-specific U5MRs in-depth. Finally, the reporting system does not include more specific causes of death; thus, targeted suggestions are difficult to conclude.

## Conclusions

The UI-specific U5MRs significantly declined in Sichuan province from 2009 to 2017, but disparities in UI-specific U5MRs in urban and rural areas still exist. Reduction of U5MRs due to UI should be a major public health concern in western China.
